# Pregnancy after the Manchester–Fothergill procedure: a case report

**DOI:** 10.1097/RC9.0000000000000452

**Published:** 2026-09-09

**Authors:** Zinat Ghanbari, Tahereh Eftekhar, Maryam Deldar, Hawraa Shbeeb, Shahla Pouyamoghadam, Marjan Ghaemi

**Affiliations:** a Department of Obstetrics and Gynecology, Family Health Research Institute, Imam Khomeini Hospital Complex, Vali-e-Asr Hospital, Tehran University of Medical Sciences, Tehran, Iran; b Department of Obstetrics and Gynecology, Vali-E-Asr Reproductive Health Research Center, Family Health Research Institute, Imam Khomeini Hospital Complex, Tehran University of Medical Sciences, Tehran, Iran

**Keywords:** cervical elongation, cervical incompetence, cesarean section, Manchester–Fothergill procedure, pregnancy, uterine prolapse

## Abstract

**Introduction and importance:**

Cervical elongation is commonly associated with uterine prolapse. The Manchester–Fothergill procedure offers a fertility-preserving surgical option for its correction. However, conception following this procedure is exceedingly rare, with an estimated incidence of 1 in 10 000.

**Case presentation:**

We present the case of a 35-year-old multiparous woman (G3P2L2) who conceived 18 months after undergoing the Manchester–Fothergill procedure for cervical elongation. Her obstetric history included one vaginal delivery and one cesarean section performed for cervical elongation. Postoperatively, she resumed regular menstrual cycles and later experienced an unplanned pregnancy.

**Clinical discussion:**

The patient was managed with close antenatal surveillance, including serial sonographic evaluations of the cervical length and fetal well-being. She carried the pregnancy to term and delivered a healthy neonate via cesarean section at 39 weeks, with a birth weight of 3,700 grams. No evidence of prolapse recurrence was observed in the postpartum period.

**Conclusion:**

This case highlights that, although rare, a successful pregnancy is possible following the Manchester–Fothergill procedure. Optimal outcomes depend on vigilant antenatal care, regular cervical monitoring, limitation of physical exertion, and early identification of potential complications.

## Introduction

Uterine prolapse is frequently associated with cervical elongation^[^1^]^. For women desiring uterine preservation, several surgical options are available, including the Manchester repair, abdominal uterosacral hysteropexy, and vaginal sacrospinous hysteropexy^[^1,2^]^. The Manchester–Fothergill procedure, originally introduced by Archibald Donald in 1888 and later refined by W.E. Fothergill, was specifically designed to treat uterovaginal prolapse in the context of cervical elongation^[^2^]^. As a uterus-conserving intervention, it offers a viable alternative to hysterectomy, particularly for younger women seeking to maintain fertility^[^3^]^. Despite its advantages, pregnancy following this procedure is exceedingly rare, with an estimated incidence of 1 in 10 000 cases^[^4^]^. Reported obstetric complications include preterm labor, spontaneous miscarriage, fetal loss, abnormal uterine bleeding, maternal urinary dysfunction, and cervical neoplasia^[^4^]^. Given these risks, close antenatal surveillance is essential for managing pregnancies following this intervention. We present a rare case of a successful term pregnancy occurring 18 months after the patient underwent the Manchester–Fothergill procedure. Recent case reports and small series have documented the possibility of successful conception and term delivery following uterus-sparing surgeries, such as the Manchester–Fothergill procedure, although the literature remains sparse. In particular, a 2022 case by Baldota *et al* highlighted a favorable maternal outcome post-embolization for hemorrhage following a modified Manchester repair, underscoring both the complexity and potential of post-surgical fertility^[^3^]^. Ertandri and Ermawati^[^4^]^ also reported a successful pregnancy outcome, emphasizing the importance of vigilant antenatal monitoring. These emerging reports offer valuable context for understanding the reproductive outcomes following conservative pelvic organ prolapse (POP) repair.

HIGHLIGHTS

- Pregnancy following the Manchester–Fothergill procedure is rare but possible.
- In order to guarantee a successful pregnancy, it is imperative to closely monitor the fetus and cervical length.
- With careful obstetric management, favorable maternal and neonatal outcomes can be achieved despite the elevated risks of preterm labor, pregnancy loss, and cervical incompetence.

## Case presentation

### Case history and examination

This case report is presented in accordance with the SCARE 2025 criteria ^[^2^]^. A 35-year-old woman, G3P2L2, presented with a positive β-hCG test at 5 weeks and 4 days' gestation.

Her obstetric history included a spontaneous vaginal delivery during her first pregnancy, 14 years earlier. One year after childbirth, she developed cervical elongation but did not seek any medical intervention. Her pelvic organ prolapse quantification (POP-Q) examination findings were (Table 1)

**Table 1:** Patient pelvic organ prolapse quantification (POP-Q) examination findings.

Anterior Wall (Aa)	Anterior Wall (Ba)	Cervix or Cut-off (C)
−2	−2	+2
Genital Hiatus (Gh)	Perineal Body (Pb)	Total Vaginal Length (TVL)
4 cm	4 cm	
		9 cm
Posterior Wall (Ap)	Posterior Wall (Bp)	Posterior Fornix (D)
−1	−1	−3

During her second pregnancy 11 years earlier, she underwent a cesarean section at 39 weeks due to cervical elongation, delivering a healthy male neonate weighing 3600 grams. Five years later, she underwent the Manchester–Fothergill procedure along with anterior and posterior colporrhaphy, perineorrhaphy, and enterocele repair (Fig. 1). Her postoperative course was uneventful, and she resumed regular menstrual cycles. She conceived unintentionally 18 months after surgery.

**Figure 1.**
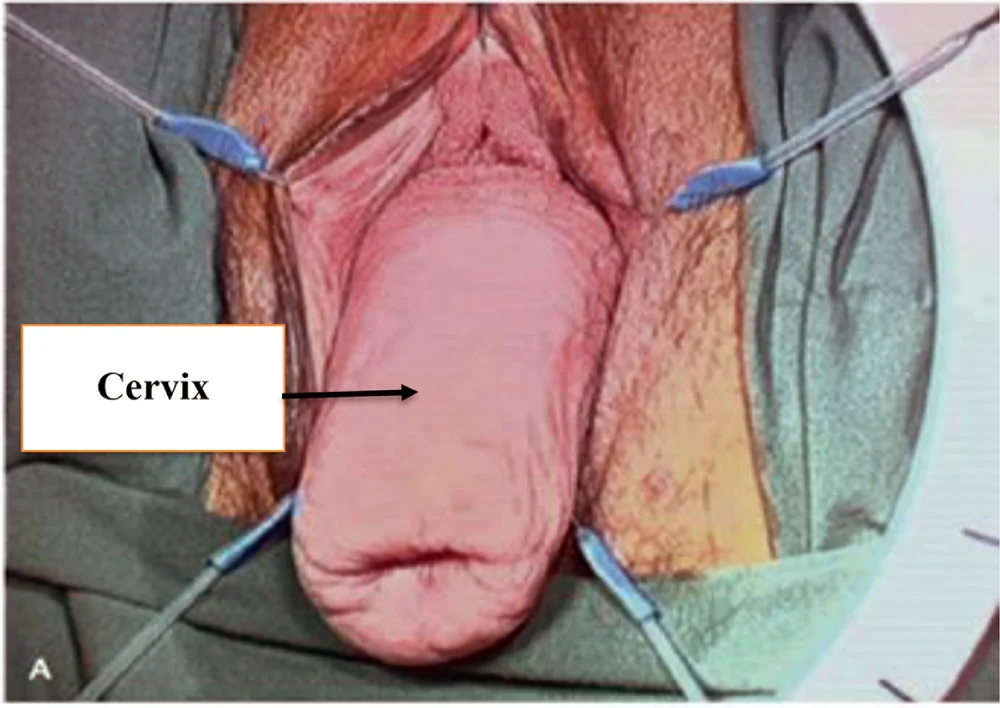
A surgical photograph demonstrates cervical elongation, necessitating surgical intervention in the form of a Manchester–Fothergill procedure.

### Methods (differential diagnosis, investigations, and treatment)

The patient was followed closely throughout gestation. Serial transvaginal ultrasonography was performed to monitor fetal growth and assess cervical length. Cervical length was consistently measured at 30 mm, with no evidence of funneling or shortening. Given the stable cervical integrity, neither cerclage placement nor progesterone therapy was indicated. No signs of prolapse recurrence were observed during physical examinations. The patient was advised to avoid excessive physical exertion. Regular antenatal visits and ultrasound evaluations were maintained throughout the pregnancy.

### Conclusions and results (outcome and follow-up)

The pregnancy progressed uneventfully, with cervical length remaining stable. At 39 weeks, an elective cesarean section was performed, resulting in the delivery of a healthy male infant (3700 grams). The decision for elective cesarean delivery at 39 weeks was made based on the potential risk of cervical trauma and the theoretical risk of prolapse recurrence associated with vaginal delivery. Intraoperatively, no evidence of uterine adhesions, cervical abnormalities, or pelvic organ prolapse was observed. Postpartum follow-up at 6, 12, 18, and 22 months revealed no recurrence of pelvic organ prolapse or menstrual irregularities. The patient reported satisfaction with the outcome and expressed gratitude for the uterus-preserving surgical option that allowed for an unexpected but successful pregnancy. This case demonstrates that pregnancy after the Manchester–Fothergill procedure can have a favorable outcome with proper antenatal surveillance. The clinical timeline of events, from the initial obstetric history to postpartum follow-up, is shown in Table 2.

**Table 2:** Visual timeline summarizing clinical history and pregnancy course.

Event	Timeline
First pregnancy (vaginal delivery)	Age 21
Second pregnancy (cesarean for cervical elongation)	Age 24
Manchester–Fothergill procedure	Age 29
Conception post-surgery	Age 30 years and 6 months
Antenatal surveillance (serial USG, no cerclage)	Weeks 6–39
Elective cesarean delivery	Week 39
Postpartum follow-up (no recurrence)	6–22 months

## Discussion

Uterine prolapse is often linked to cervical elongation and is typically treated through surgery, especially in women seeking to maintain their fertility. The Manchester–Fothergill method, initially outlined by Donald and subsequently revised by Fothergill, continues to be a suitable fertility-preserving alternative to hysterectomy for certain patients^[^5^]^. Nonetheless, conceiving after this procedure is extremely uncommon, with an estimated rate of 1 in 10 000 cases^[^4^]^.

The main concern in pregnancies following a Manchester procedure is cervical insufficiency, which can result in negative obstetric outcomes, such as preterm labor and pregnancy loss during the second trimester^[^6^]^. Certain studies indicate that the rate of pregnancy loss in such cases may reach up to 50%^[^4^]^. Additional complications reported include cervical stenosis, hematometra, pyometra, and recurrence of postpartum prolapse^[^7^]^. Due to these associated risks, diligent monitoring is crucial to enhance both maternal and neonatal outcomes.

Management approaches for pregnancies following a Manchester procedure primarily aim to preserve cervical competence and prevent preterm labor. Regular sonographic monitoring of cervical length is essential for risk assessment, with a cervical length of <25 mm serving as a critical threshold for intervention^[^6^]^. In certain instances, cervical cerclage may be considered; however, it was not necessary in our patient due to a stable cervical length of 30 mm. Furthermore, progesterone supplementation has been proposed as a preventive strategy for women with cervical shortening, though it was not deemed appropriate in this case.

Delivery planning is a critical consideration in pregnancies following the Manchester procedure. Although vaginal delivery has been documented, the majority of studies advocate for elective cesarean section to mitigate the risks of cervical injury and recurrence of prolapse^[^8^]^. In the present case, an elective cesarean section performed at 39 weeks’ gestation led to a positive neonatal outcome, consistent with findings from previous studies^[^3^]^.

When compared with the few reported cases in the literature, this case aligns with key observations: stable cervical length, absence of prolapse recurrence during pregnancy, and favorable neonatal outcomes with elective cesarean delivery. Jasonni *et al*^[^8^]^ similarly documented a positive pregnancy outcome after Manchester repair with close follow-up. These findings collectively support the conclusion that, with appropriate surgical technique and diligent antenatal care, uterine conservation is compatible with future pregnancy.

Postpartum follow-up is essential for the evaluation of prolapse recurrence and menstrual irregularities. Long-term studies suggest that although prolapse recurrence remains a concern, successful pregnancies without recurrence have been documented^[^1^]^. In the case of our patient, no prolapse recurrence was observed during a 22-month follow-up period.

This case underscores that while pregnancy following the Manchester–Fothergill procedure is uncommon, it can result in favorable maternal and neonatal outcomes when appropriate antenatal surveillance is conducted. A multidisciplinary approach, involving obstetricians and urogynecologists, is recommended to optimize the management of such pregnancies and ensure positive long-term outcomes for these patients.

## Conclusion

The Manchester–Fothergill procedure is an effective uterus-sparing option for women with uterine prolapse who desire future fertility. Although rare, successful pregnancies are achievable with close monitoring, proper management, and a planned delivery. With multidisciplinary coordination, this surgical approach can result in excellent maternal and neonatal outcomes.

## Key clinical takeaways

- Individualized delivery planning is essential; a cesarean section is often the safest choice.
- Vigilant monitoring of cervical length is key to reducing the risk of preterm birth.
- Long-term follow-up is critical for assessing prolapse recurrence.
- Patients should be counseled that fertility is possible but must be considered high-risk post-surgery.

## Patient perspective

The patient expressed gratitude for the uterus-preserving surgery, stating that although she did not anticipate future fertility, the outcome was both unexpected and welcome. She reported no psychological distress during her pregnancy and appreciated the thorough antenatal care provided.

## Data Availability

Data sharing is not applicable to this article as no datasets were developed or analyzed during the study.

## Clinical key message

Pregnancy following the Manchester–Fothergill procedure is rare but possible. In order to guarantee a successful pregnancy, it is imperative to closely monitor the fetus and cervical length. With careful obstetric management, favorable maternal and neonatal outcomes can be achieved despite the elevated risks of preterm labor, pregnancy loss, and cervical incompetence.
